# The early and long-term outcomes of coronary artery bypass grafting added to aortic valve replacement compared to isolated aortic valve replacement in elderly patients: a systematic review and meta-analysis

**DOI:** 10.1007/s00380-022-02073-4

**Published:** 2022-05-09

**Authors:** Stefano D’Alessandro, Domenico Tuttolomondo, Gurmeet Singh, Daniel Hernandez-Vaquero, Claudia Pattuzzi, Alan Gallingani, Francesco Maestri, Francesco Nicolini, Francesco Formica

**Affiliations:** 1 Cardiac Surgery Unit, San Gerardo Hospital, Monza, Italy; 2 Cardiology Unit, University Hospital of Parma, Parma, Italy; 3 Department of Critical Care Medicine and Division of Cardiac Surgery, Mazankowski Alberta Heart Institute, University of Alberta, Edmonton, AB Canada; 4 Cardiac Surgery Department, Hospital Universitario Central de Asturias, Oviedo, Spain; 5 Cardiac Surgery Unit, University Hospital of Parma, Parma, Italy; 6 Department of Medicine and Surgery, University of Parma, Parma, Italy; 7 UOC Cardiochirurgia, Azienda Ospedaliera Universitaria di Parma, Via A. Gramsci, 14, 43126 Parma, Italy

**Keywords:** Aortic valve replacement, Coronary artery bypass grafting, Elderly, Long-term outcomes, Meta-analysis

## Abstract

In aged population, the early and long-term outcomes of coronary revascularization (CABG) added to surgical aortic valve replacement (SAVR) compared to isolated SAVR (i-SAVR) are conflicting. To address this limitation, a meta-analysis comparing the early and late outcomes of SAVR plus CABG with i-SAVR was performed. Electronic databases from January 2000 to November 2021 were screened. Studies reporting early-term and long-term comparison between the two treatments in patients over 75 years were analyzed. The primary endpoints were in-hospital/30-day mortality and overall long-term survival. The pooled odd ratio (OR) and hazard ratio (HR) with 95% confidence interval (CI) were calculated for in-early outcome and long-term survival, respectively. Random-effect model was used in all analyses. Forty-four retrospective observational studies reporting on 74,560 patients (i-SAVR = 36,062; SAVR + CABG = 38,498) were included for comparison. The pooled analysis revealed that i-SAVR was significantly associated with lower rate of early mortality compared to SAVR plus CABG (OR = 0.70, 95% CI 0.66–0.75; *p* < 0.0001) and with lower incidence of postoperative acute renal failure (OR = 0.65; 95% CI 0.50–0.91; *p* = 0.02), need for dialysis (OR = 0.65; 95% CI 0.50–0.86; *p* = 0.002) and prolonged mechanical ventilation (OR = 0.57; 95% CI 0.42–0.77; *p* < 0.0001). Twenty-two studies reported data of long-term follow-up. No differences were reported between the two groups in long-term survival (HR = 0.95; 95% CI  0.87–1.03; *p* = 0.23). CABG added to SAVR is associated with worse early outcomes in terms of early mortality, postoperative acute renal failure, and prolonged mechanical ventilation. Long-term survival was comparable between the two treatments.

**Supplementary Information:**

The online version contains supplementary material available at 10.1007/s00380-022-02073-4.

## Introduction

The elderly population continues to increase in Europe and United States (US), especially age greater than 75 years. This group is expected to grow considerably over the next 20 years. In Europe, the population over 75 years is expected to reach 65 million by 2040, an approximately 49% increase compared to 2020 [1]. By 2040 in the United States, the population over age 75 is expected to rise from about 23 million today, to more than 43 million, a projected increase of about 90% [2, 3]. Because aortic valve stenosis (AS) and coronary artery disease (CAD) are the most commonly represented cardiac lesions in the elderly, as the elderly population increases a concomitant rise in AS and CAD is anticipated. Advanced age is associated with considerable number of comorbidities and medical frailty, exposing the elderly patient to potentially considerable operative risk. Moreover, simultaneous surgical aortic valve replacement (SAVR) and coronary artery bypass grafting (CABG) carries a higher procedural risk compared with isolated SAVR (i-SAVR). Indeed, even if AS could be addressed with transcatheter aortic valve implantation (TAVI) even in patients older than 75 years with intermediate or low risk [4], the combination of TAVI and percutaneous coronary intervention (PCI) is not a widely accepted practice, especially for those patients with a recognized heavily calcified CAD. Several studies [5–10] have reported relevant early results in patients who underwent either i-SAVR or SAVR combined with CABG. Other authors [11–16] have reported unfavorable early outcome in those patients who underwent simultaneous SAVR and CABG. The results are still debated regarding long-term outcomes, as some studies have reported acceptable and comparable long-term outcomes [17, 18], whereas other authors have reported conflicting results, with some studies showing better long-term survival in i-SAVR patients [19] and others reporting better long-term outcomes in SAVR plus CABG patients [20, 21]. No randomized control trials are available and, to the best of our knowledge, no meta-analyses have addressed the impact of concomitant CABG and SAVR in elderly patients. To address this limitation, a systematic review and meta-analysis was conducted with the best available evidence, evaluating the impact on early-term and long-term outcomes of CABG combined with SAVR, compared to i-SAVR in patients greater than 75 years of age.

## Materials and methods

### Systematic review of the literature, search strategy and eligibility criteria

A comprehensive review of previous relevant studies which were published from 1 January 2000 to 30 November 2021 was conducted. The search was conducted using the electronic databases PubMed and EMBASE. Search terms used alone or in combination included “elderly patients,” “very elderly,” “octogenarians,” “surgical aortic valve replacement,” “coronary artery bypass grafting,” “early-term results,” “75 years old” and “80 years old.” Furthermore, the references list of the obtained articles was used to implement the search.

The literature search and review were based on the PICOS format (Population; Intervention; Comparison; Outcomes; Studies); *Population*: patients with isolated aortic valve disease or combined with coronary artery disease; *Intervention*: i-SAVR; Comparison: SAVR plus CABG; *Outcomes*: early and long-term outcomes; *Studies*: randomized trials, retrospective and prospective observational studies.

Selection of relevant studies was conducted according to the following inclusion criteria: (1) patients who underwent either i-SAVR or SAVR in addition to CABG; (2) patients older than 75 years; (3) early mortality comparing the two surgical interventions; (4) long-term survival comparing the two operations; (5) studies included any of the following postoperative complications: atrial fibrillation (POAF), acute renal failure, need for dialysis, pneumonia, prolonged mechanical ventilation (PMV), stroke, re-thoracotomy for bleeding/tamponade, need for postoperative intra-aortic balloon pump (IABP), length of stay and early mortality. Studies including in the analysis other associated cardiac procedure were excluded. Moreover, studies which were published in languages other than English were excluded, as were commentaries, letters, case reports, systematic reviews and meta-analyses.

This systematic review and meta-analysis were conducted according to the Preferred Reporting Items for Systematic Reviews and Meta-Analyses (PRISMA) guidelines [22] and was based on the following steps: (1) identification of titles and abstracts of records through database search; (2) removal of duplicates; (3) screening and selection of abstracts; (4) evaluation of study eligibility through full-text articles; and (5) final inclusion in study. Studies were selected by two independent authors (SDA, DT). When there was disagreement, a third senior author (FF) reviewer made the decision of whether to include or exclude the study.

The study protocol of the systematic review and meta-analysis was registered and published online in PROSPERO (The International Prospective Register of Systematic Reviews; ID: CRD42021276831).

### Data extraction and database

Two reviewers (SDA and DT) independently performed data extraction which were reported in a standard table sheet database (Microsoft Office Excel 2016, Microsoft, Redmond, WA, USA). Median and interquartile ranges were converted into mean and standard deviations following the recommendations of Luo et al. [23]. All studies included in the meta-analysis were identified by first author, country, study design, study period, and year of publication. The following patient factors were collected: age, gender (male), POAF, postoperative acute renal failure, need for dialysis, postoperative pneumonia, PMV, postoperative stroke, re-thoracotomy for bleeding/tamponade, postoperative IABP, postoperative length of stay.

### Endpoints

The primary endpoints of the meta-analysis were the (i) early mortality, defined as death occurred within 30 days or during the index admission and (ii) the overall long-term survival. The secondary endpoints were the following postoperative complications: new onset of POAF, renal failure, need for dialysis, pneumonia, PMV (> 48 h), any stroke, re-thoracotomy for bleeding, need for IABP and length of stay.

### Statistical analysis

The pooled effect size with odd ratio (OR) and 95% Confidence Interval (CI) using the Mantzel–Haenszel method was calculated for early mortality and for the secondary endpoints. The pooled hazard ratio (HR) with 95% CI using the Mantzel–Haenszel method was calculated for long-term survival. The random-effect model was preferred because the variability across the studies was taken into account in the model. HR and the corresponding 95% CI was calculated analysing time-to event outcomes according to the methods proposed by Tierney et al. [24]. When available, the reported HRs of selected studies were compared with the estimated HRs.

Weighted mean differences were calculated for the continuous variable length of stay. Forest plots were created to represent the primary and secondary outcomes and to determine the effect size. Statistical heterogeneity was assessed with Chi-square test and *I*^2^ test and defined as low for *I*^2^ ranging from 0% to 25%, moderate for *I*^2^ ranging from 26% to 50% and high for *I*^2^ above 50% [25]. Publication bias was assessed for each endpoint by generating the funnel plots using the trim and fill method and analysed by means of Egger’s test and Begg and Mazumdar’s test and estimated visually. Possible publication bias was suggested also by asymmetric funnel plot. Sensitivity analysis was applied to verify the influence of a single study on the primary endpoints, by sequentially removing one study, according to the leave-one-out method [26].

Categorical variables were reported as number and percentages. Continuous variables were reported as mean and standard deviation. A two-tailed *p*-value < 0.05 was considered to indicate statistical significance. All statistical analyses were completed with ProMeta3 software (http://idostatistics.com/prometa3/), and with the Review Manager (RevMan5) Version 5.3 (The Cochrane Collaboration, 2012, The Nordic Cochrane Centre and Copenhagen, Denmark).

## Results

A total of 2046 titles and abstracts were identified, of which 57 were considered potentially relevant and for the meta-analysis and retrieved as full-text. After evaluating the full-text articles, 44 studies [5–21, 27–53] fulfilled the eligibility criteria and were included in the final analysis. All included studies were observational and retrospective in design, an no randomized clinical trials or prospective studies were identified. The PRISMA Flow Chart of study selection process is shown in Supplemental Fig. 1.

A total of 74,560 patients were extracted from the selected articles. I-SAVR included 36,062 patients (48.5%) and SAVR plus CABG included 38,498 patients (51.5%). Characteristics of studies, and preoperative data of patients included in each study are shown in Table 1. Postoperative data are listed in Table 2.

**Table 1:** Study typology and patient’s baseline characteristics

First author	Year	i-SVAR (*n* = 36,477)	SAVR + CABG (*n* = 38,741)	Male gender (%)	Age (mean)	EF (mean)	Hypertension (%)	Diabetes (%)	Stroke (%)	RF (%)	COPD (%)	PAF (%)
Thullin	2000	121	98	39	78	–	–	6.8	3.6	–	–	–
Ennker	2001	62	52	27.2	82.8	–	51.4	22	5.3	21.6	8	22
Nikolaidis	2001	161	184	–	82.9	–	–	6.9	4.9	3.4	11.6	–
Brunvald	2002	42	52	35	82	70	–	–	–	–	–	–
Lithmate	2003	166	117	55	–	–	–	–	–	–	–	–
Chiappini	2004	71	44	54	82.3	–	–	13.8	–	–	–	15.5
Lam	2004	30	28	62	83.7	40	35	12		9	7	3
Unic	2005	94	146	48	83	–	55	12	–	12	12	–
Bose	2007	37	31	58	83.2	–	43	–	–	9	18	–
Kolh	2007	162	58	21	82.8	–	41	12	–	4	5	14
Melby	2007	140	105	53	83.6	–	69	18	–	8	12	
Ngaage	2007	98	89	–	–	–	–	–	–	–	–	–
Roberts	2007	78	118	–	–	–	–	–	–	–	–	–
Urso	2007	66	34	52	82.1	–	56	9	–	17		19
Huber	2007	34	41	54.6	82.5	–	60	10.6	–	21.3	18.6	25.3
Filsoufi	2008	82	110	–	–	–	–	–	–	–	–	–
Likosky	2009	575	815	49.6	–	–	–	16.7	–	4.1	6	–
Maillet	2009	49	35	37	83.7	59	–		–	16.7	22.6	26.2
Florath	2010	252	241	32	83	53	71	29	9	23	27	19
Folkmann	2010	74	80	33.8	82.9	–	–	30.3	–	38.7	54.9	
Maslow	2010	145	116	54.4	83	51	78.2	22.6	–	9.2	13.8	3.1
Dell'Amore	2011	188	97	61.7	82	–	77.9	45.6	9.1	7.3	14.7	20.3
Kesavan	2011	140	133	47	82.7	–	–	11	15	4	17	–
Krane	2011	303	297	39.5	82.5	–	80.1	20.6	–	–	–	24
Yamane	2011	68	56	52	83.5	50.9	70	21	15	5.6	19	
Langanay	2012	883	310	48	82.5	59.5	–	8	–	10	13	13
Abel	2013	117	263	48.1	83	–	74.5	26.8	–	9.7	12	–
Harris	2013	60	57	53.8	83	–	71.8	15.4	14.5	5.1	13.7	–
Mitchell	2013	46	21	–	–	–	–	–	–	–	–	–
Raja	2013	68	114	64.8	82	–	80.2	38.5	14.2	35.5	36.6	22
Sasaki	2013	120	37	76.5	43.9	57	65.6	31.2		8.9	–	–
Budniak	2014	28	12	62.5	81	–	–	25	5	35	7.5	15
Davis	2014	38	25	–		–	–	–	–	–	–	–
Grau	2014	88	105	58.2	75	–	86	31.7	6	2	14.2	–
Ho	2014	49	83	56	83	–	69	23	18	8	14	–
Kamiya	2014	179	159	38.7	81.5	58	87.6	29.6			24	–
Agarwal	2015	29,343	32,449	–	–	–	–	–	–	–	–	–
Salsano	2016	32	23	45.6	86	52	57.8	10.5	3.5	3.5	10.5	
Wang	2016	93	104	64	83	–	57.3	11.2	5.6	1	23.3	28.5
Kuo	2017	170	208	58.1	83	–	67.5	19.6	5.8	–	11.4	18.8
Ennker	2018	317	404	40.6	83	–	78.8	25.3	1 + S36	–	–	19
Dimagli	2020	935	777	–	–	–	–	–	–	–	–	–
Formica	2020	200	202	48	78.1	55.4	81.6	23.4	8	38.6	13.4	17
Takagi	2020	18	11	17.2	87	–	86	20.7	20.7	10	13.8	6.9

*SAVR* surgical aortic valve replacement; *CABG* coronary artery bypass grafting; *CVE* cerebrovascular events; *RF* renal failure; *COPD* chronic obstructive pulmonary disease; *PAF* permanent atrial fibrillation; *EF* ejection fraction

**Table 2:** Postoperative data

Authors	Re-thoracothomy for bleeding (%)	IABP (%)	CVE ( %)	AF (%)	RF (%)	Dialysis (%)	Mortality i-SAVR, *n* (%)		Mortality SAVR + CABG, *n* (%)	
Thullin	–	–	–	–	–	–	1	0.8	4	4.1
Ennker	5	5.5	0.9	45	4	–	3	4.8	8	15.4
Nikolaidis	–	1.7	2	–	8.9	–	9	5.6	11	6.0
Brunvald	–	–	–	–	–	–	4	9.5	4	7.7
Lithmate	–	–	–	–	–	–	7	4.2	6	5.1
Chiappini	–	–	0.8	17.3	–	–	3	4.2	7	15.9
Lam	14	–	5	48	19	–	2	6.7	3	10.7
Unic	–	–	–	–	–	–	4	4.3	12	8.2
Bose	4	–	1	26	–	10	4	10.8	5	16.1
Kolh	4	–	2	24	–	5	15	9.3	14	24.1
Melby	9	5	3	45	12	–	11	7.9	7	6.7
Ngaage	–	–	–	–	–	–	5	5.1	7	7.9
Roberts	–	–	–	–	–	–	8	10.3	13	11.0
Urso	–	–	3	35	26	–	6	9.1	2	5.9
Huber	5.3	0	4	28	–	–	1	2.9	3	7.3
Filsoufi	–	–	–	–	–	–	5	6.1	3	2.7
Likosky	5.6	–	3	44.5	–	–	45	7.8	84	10.3
Maillet	7.1	–	5.9	45.2	–	11.9	5	10.2	9	25.7
Florath	–	–	–	–	–	–	19	7.5	23	9.5
Folkmann	–	–	–	–	–	–	5	6.8	7	8.8
Maslow	4.6	–	3.1	31	8.4	2.7	8	5.5	8	6.9
Dell'Amore	6	2.1	2.1	37.5	14	2.5	8	4.3	7	7.2
Kesavan	5	–	5	–	16	–	8	5.7	11	8.3
Krane	4.6	–	2.3	27.5	12.1	–	24	7.9	30	10.1
Yamane	4	8	2.4	39	4.8	–	3	4.4	2	3.6
Langanay	3.4	–	1.7	47	10.4	1	48	5.4	36	11.6
Abel	–	–	–	36	11.3	3.7	6	5.1	20	7.6
Harris	9.4	–	0	22.2	6	4.3	2	3.3	2	3.5
Mitchell	–	–	–	–	–	–	0	0.0	1	4.8
Raja	–	–	–	–	–	–	5	7.4	11	9.6
Sasaki	6.3	–	3.1	–	–	–	1	0.8	2	5.4
Budniak	–	–	2.5	–	15	7.5	0	0.0	0	0.0
Davis	–	–	–	–	–	–	7	18.4	3	12.0
Grau	–	–	2	24.7	3	–	1	1.1	3	2.9
Ho	12.9	–	6	24	7.6	–	3	6.1	8	9.6
Kamiya	8.3	1.8	0.9	–	–	9.7	15	8.4	19	11.9
Agarwal	–	–	3.3	–	–	–	1210	4.1	1826	5.6
Salsano	21	–	1.7	–	6	3.2	1	3.1	4	17.4
Wang	8.6	–	3.5	–	2	–	0	0.0	7	6.7
Kuo	13.8	–	–	–	14.6	2.7	12	7.1	14	6.7
Ennker	–	–	1.8	–	–	12	32	10.1	54	13.4
Dimagli	–	–	–	–	–	–	23	2.5	43	5.5
Formica	6	2	3.2	45.5	7.5	2.2	9	4.5	13	6.4
Takagi	3.4	–	3.4	–	–	–	0	0.0	0	0.0

*SAVR* surgical aortic valve replacement; *CABG* coronary artery bypass grafting; *CVE* cerebrovascular events; *RF* renal failure; *AF* atrial fibrillation; *IABP* intra-aortic balloon pump

### Primary endpoints: early mortality and long-term survival

**Fig. 1:**
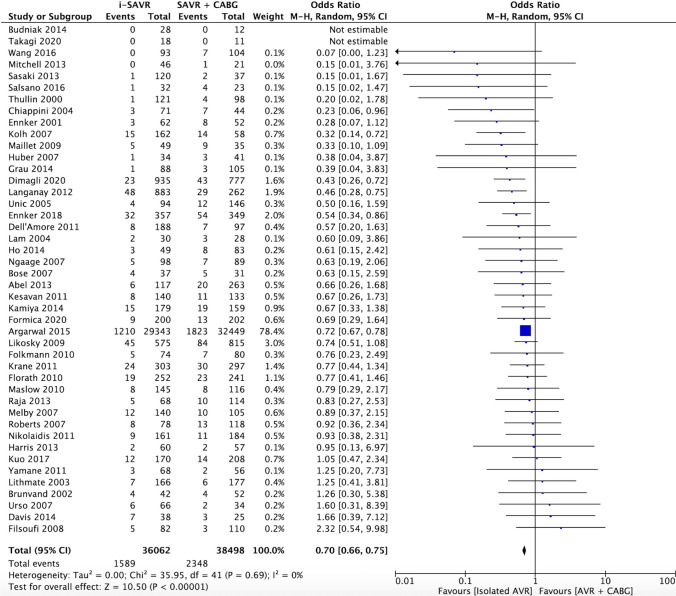
Forest plot of comparison for early mortality. *I-SAVR* isolated surgical aortic valve replacement; *CABG* coronary artery bypass grafting; *M*–*H* Mantzel–Haenszel

All studies included in the meta-analysis reported the early mortality comparison between i-SAVR and SAVR combined with CABG. The pooled analysis revealed a significant difference between the two groups, favoring i-SAVR treatment (OR 0.70, 95% CI 0.66–0.75; *p* < 0.0001) with no evidence of heterogeneity (*I*^2^ = 0%, Tau^2^ = 0.01, *p* = 0.69) (Fig. 1). The leave-one-out analysis did not identify any influential studies on the aggregated data, with each study removed each time and the meta-analysis repeated n times the number of studies included in the analysis. (Supplemental Fig. 2A). Funnel plot analysis did not reveal asymmetry around the axis, with no evidence of publication bias (Egger’s linear regression test: *p* = 0.06; Begg and Mazumdar’s test: *p* = 0.15) (Supplemental Fig. 3A).

**Fig. 2:**
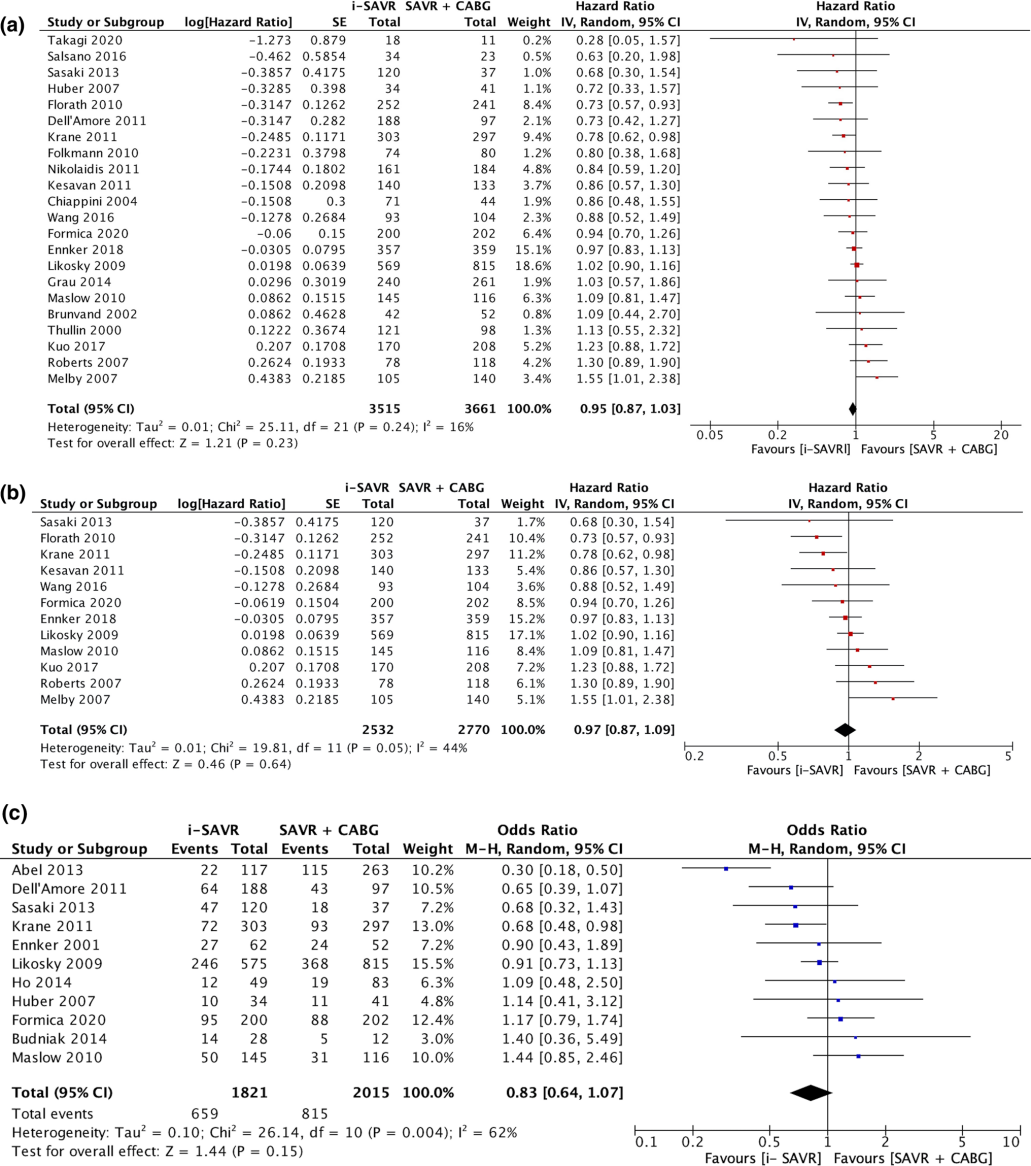
**A**. Forest plot of comparison for long-term survival. **B**. Forest plot of comparison for long-term survival of studies reporting a maximum follow-up of 10 years or more. **C**. Forest plot for new onset of postoperative atrial fibrillation. *I-SAVR* isolated surgical aortic valve replacement; *CABG* coronary artery bypass grafting; *M*–*H* Mantzel–Haenszel

Twenty-two studies [5, 7, 9, 11, 13, 17–21, 27, 29, 34, 38, 39, 41, 45, 50–53] reported long-term survival comparison between the two surgical interventions with a mean follow-up ranging from 2.1 years [27] to 9.5 years [45]. The weighted mean follow-up was 3.2 years. The longest follow-up was 16.1 years [10]. The pooled analysis of long-term survival did not reveal difference between the two treatments (HR = 0.95; 95% CI 0.87–1.03; *p* = 0.23) with evidence of low heterogeneity (*I*^2^ = 16%; Tau^2^ = 0.01; *p* = 0.24) (Fig. 2A).

The leave-one-out analysis did not identify any influential studies on the pooled data. (Supplemental material, Fig. 2B). No evidence of publication bias was found assessed by the Egger’s linear regression test (*p* = 0.27) and Begg and Mazumdar’s linear regression test (*p* = 0.19) or by visual inspection of the funnel plot (Supplemental material, Fig. 3B). When we restricted the analysis for those studies (*n* = 12) [7, 9, 10, 13, 19, 20, 38, 39, 41, 45, 51, 52] reporting a maximum follow-up of 10 years or more, the pooled analysis revealed no differences between the two groups (HR = 0.97; 95% CI 0.87–1.09; *p* = 0.64) with evidence of moderate heterogeneity (*I*^2^ = 44%; Tau^2^ = 0.01; *p* = 0.05) (Fig. 2B) and with no evidence of publication bias (Egger’s linear regression test: *p* = 0.83; Begg and Mazumdar’s test: *p* = 0.90) (Supplemental material, Fig. 3C). The weighted mean follow-up and was 5.3 years.

### Secondary endpoints

**Fig. 3:**
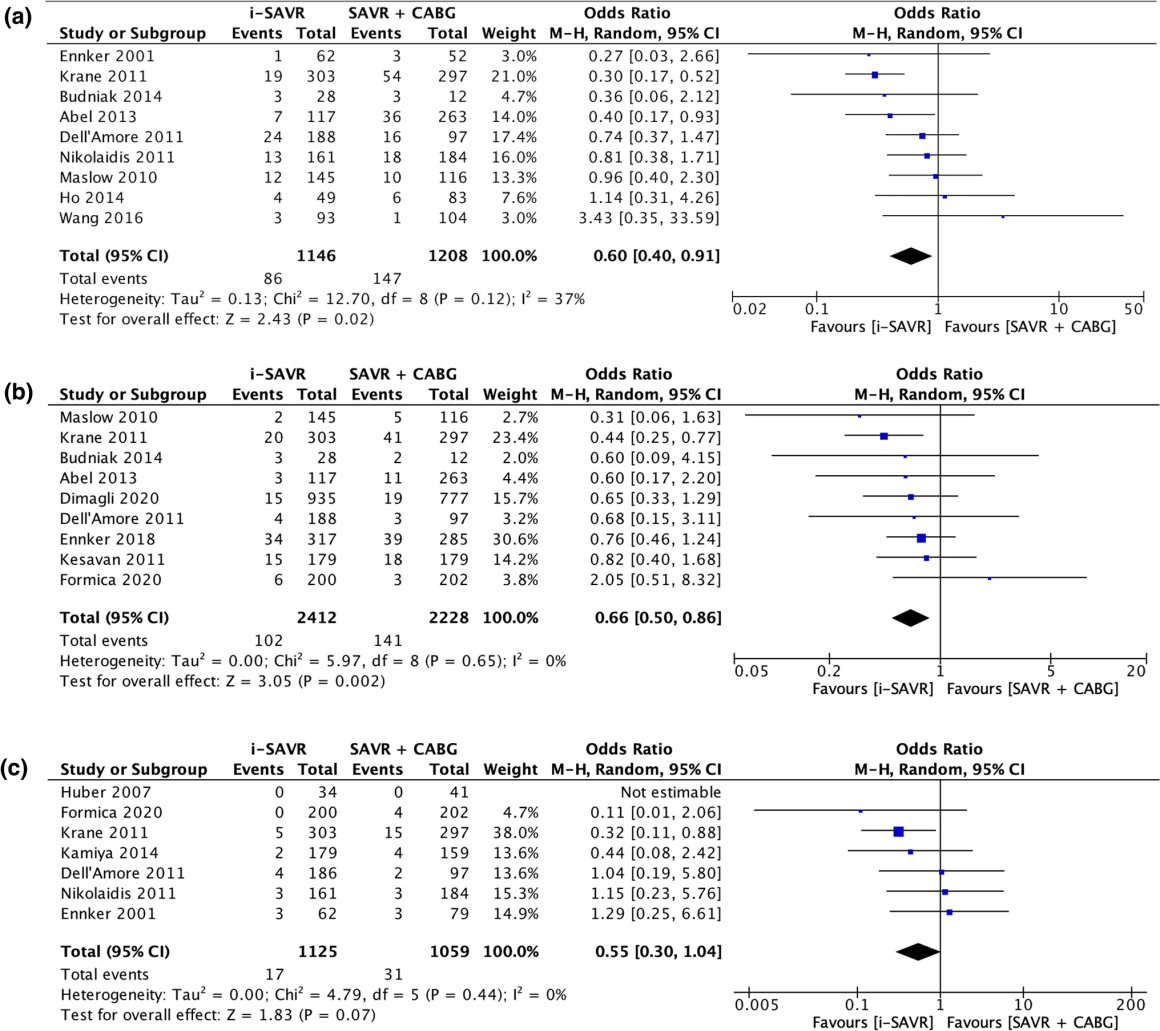
**A** Forest plot for new onset of postoperative acute renal failure. **B** Forest plot for postoperative need for dialysis. **C** Forest plot for postoperative intra-aortic balloon pump. *I-SAVR* isolated surgical aortic valve replacement; *CABG* coronary artery bypass grafting; *M*–*H* Mantzel–Haenszel

**Fig. 4:**
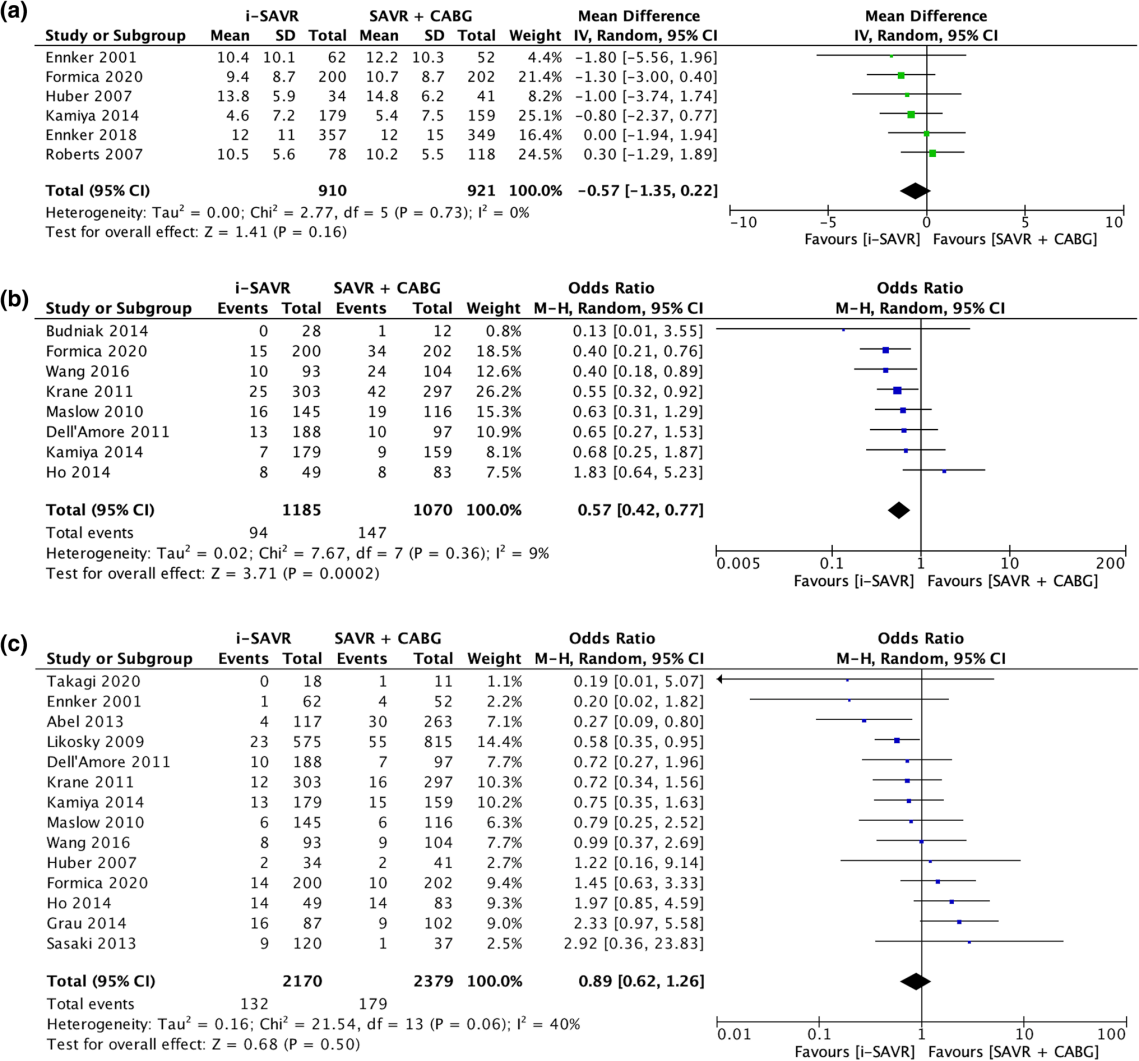
**A** Forest plot for length of hospital stay. **B** Forest plot for prolonged mechanical ventilation. **C** Forest plot for re-thoracotomy for postoperative bleeding/tamponade. *I-SAVR* isolated surgical aortic valve replacement; *CABG* coronary artery bypass grafting; *M*–*H* Mantzel–Haenszel

**Fig. 5:**
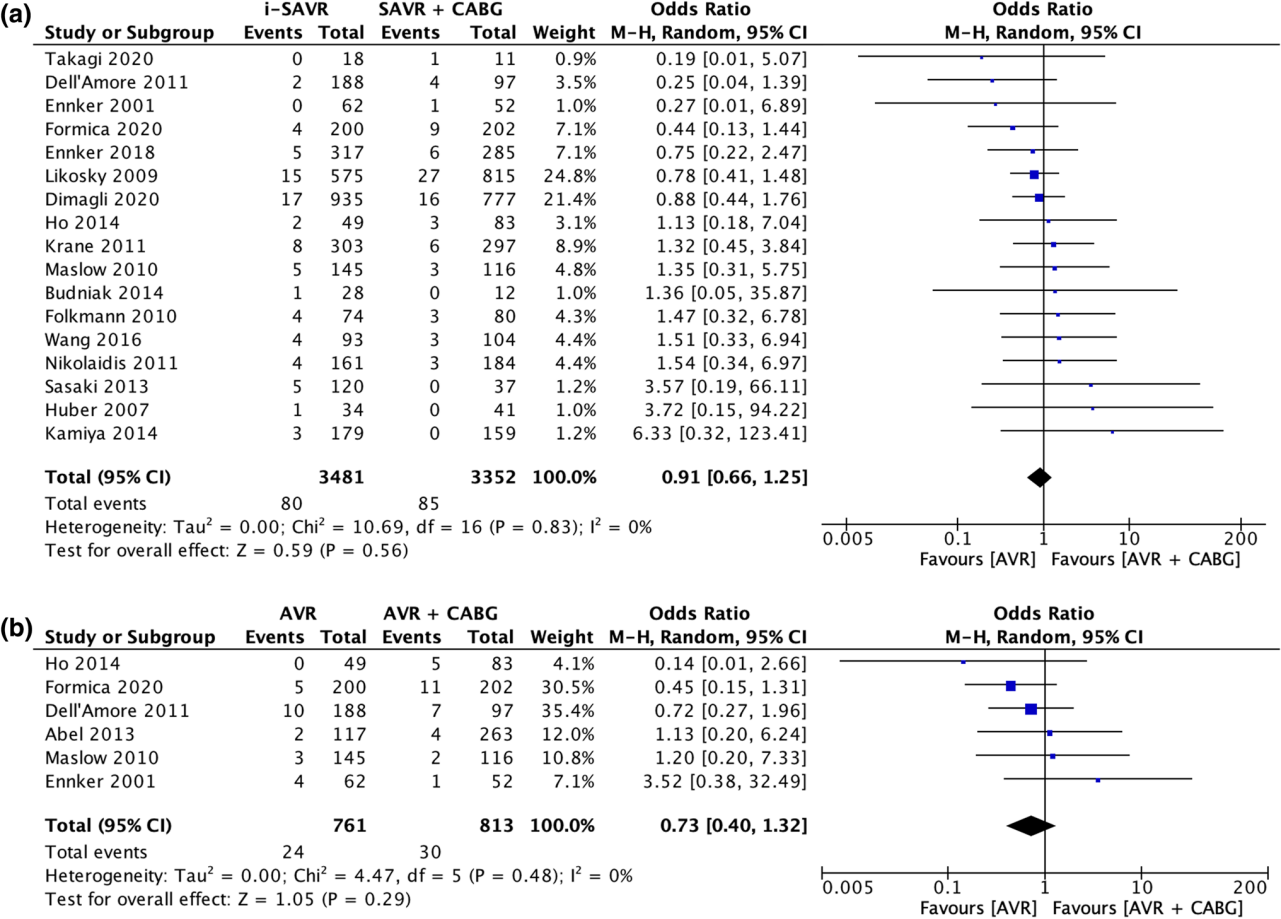
**A** Forest plot for postoperative stroke. **B** Forest plot for postoperative pneumonia. *I-SAVR* isolated surgical aortic valve replacement; *CABG* coronary artery bypass grafting; *M*–*H* Mantzel–Haenszel

**Fig. 6:**
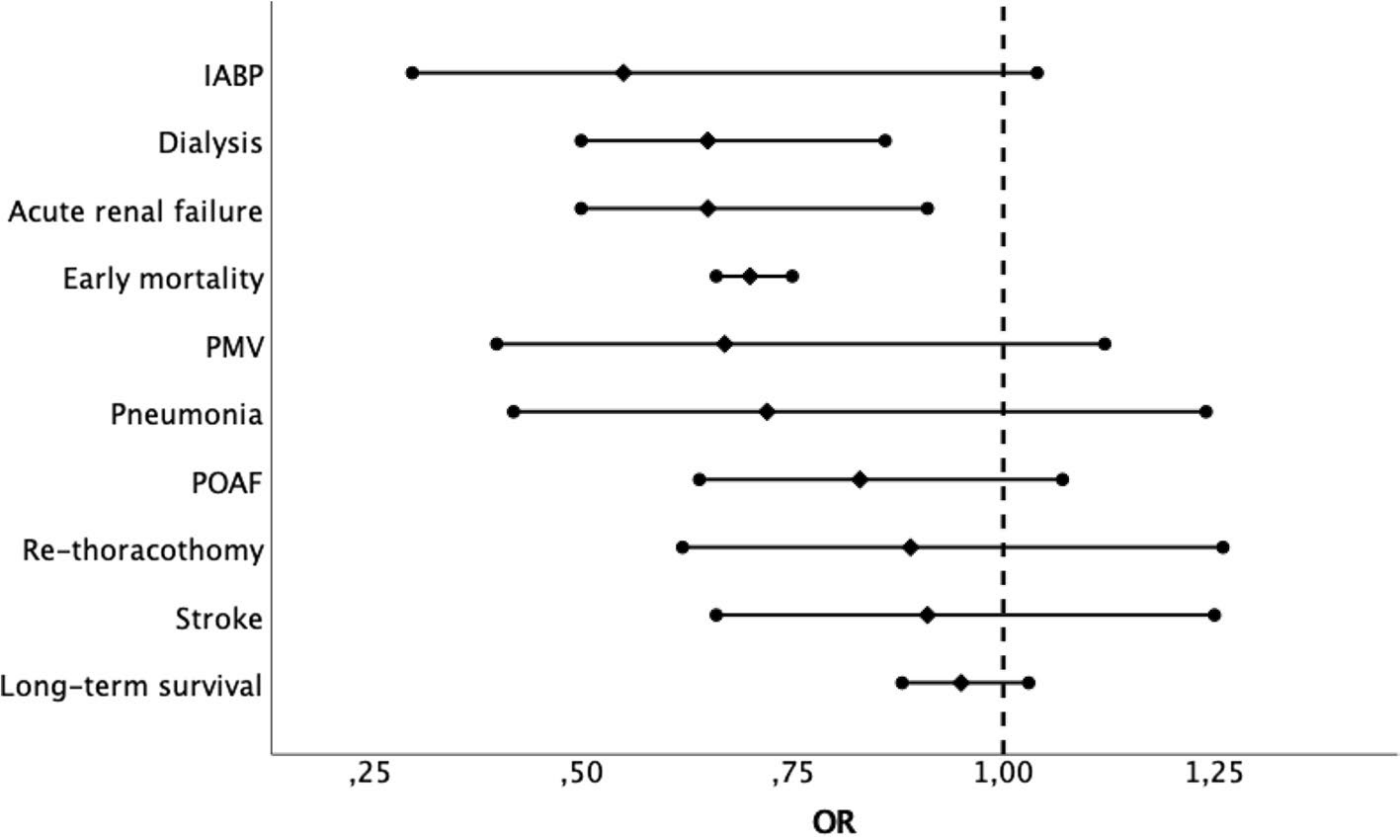
Cumulative forest plot of primary and secondary end-points. *IABP* intra-aortic balloon pump; *AKI* acute kidney injury; *PMV* prolonged mechanical ventilation; *POAF* postoperative atrial fibrillationation

The odds of postoperative atrial fibrillation were comparable between the two groups (OR = 0.83; 95% CI 0.64 –1.07; *p* = 0.15) with significant heterogeneity (*I*^2^ = 62%) (Fig. 2C). Postoperative acute renal failure incidence was reduced in patients received i-SAVR compared to those received additional CABG (OR = 0.60; 95% CI 0.40–0.91; *p* = 0.02), with evidence of moderate heterogeneity (*I*^2^ = 37%) (Fig. 3A). Similarly, reduced odds of postoperative dialysis were more represented in patients who underwent i-SAVR compared to SAVR + CABG (OR = 0.66; 95% CI 0.50–0.86; *p* = 0.002), with no evidence of heterogeneity (*I*^2^ = 0%) (Fig. 3B). I-SAVR group had a nonsignificant reduced odds of postoperative IABP usage (OR = 0.55; 95% CI 0.30–1.04; *p* = 0.07), with no heterogeneity (*I*^2^ = 0%) (Fig. 3C). No significant differences were found regarding length of postoperative hospital stay (mean difference = −0.57; 95% CI −1.35–0.22; *p* = 0.16; *I*^2^ = 0%) (Fig. 4A). Reduced odds of PMV were observed in patients who underwent i-SAVR (OR = 0.67; 95% CI 0.40–1.12; *p* < 0.13) with high heterogeneity (*I*^2^ = 67%) (Fig. 4B). No differences between the two operations were observed regarding re-thoracotomy for bleeding/tamponade (OR = 0.89; 95% CI 0.62–1.26; *p* = 0.50; *I*^2^ = 40%) (Fig. 4C), postoperative stroke (OR = 0.91; 95% CI 0.66–1.25; *p* = 0.56; *I*^2^ = 0%) (Fig. 5A) and postoperative pneumonia (OR = 0.73; 95% CI 0.40–1.32; *I*^2^ = 0%) (Fig. 5B). The pooled effect sizes are summarized in the Fig. 6. Analysis of the funnel plots showed symmetry and no evidence of risk of publication bias (Supplemental Materials, Fig. 3D through 3N).

## Discussion

AS is the most frequently identified lesion in the elderly patients, with incidence increasing with age, exceeding 5% in patients over 80 years [54, 55]. Previous studies reported that almost half of the elderly patients undergoing SAVR were more likely to require CABG, compared to the non-elderly requiring SAVR [56–58].

By this comprehensive systematic review and meta-analysis, we aimed to analyze the impact of CABG in the aged population requiring SAVR, and to the best of our knowledge this is the first meta-analysis focusing on this topic. The main findings where that (i) CABG in combination with SAVR is associated with higher early mortality compare to i-SAVR, (ii) the long-term survival is comparable between the two surgical operations and (iii) CABG plus SAVR is associated with a higher rate of postoperative complications such as acute renal failure, need for dialysis, and PMV. Interestingly, the rate of new onset POAF, IABP usage, postoperative stroke, re-thoracotomy for bleeding/tamponade, postoperative pneumonia and length of hospital stay were similar in both groups.

CAD has an unfavorable prognostic factor, accentuated even further in presence of left main stenosis greater than 50%. Such patients have an increased risk of developing myocardial injury likely secondary to an imbalance between myocardial oxygen supply and demand during cardiac surgery. Previous studies demonstrated that cardiac troponin (c-Tn) levels measured after cardiac surgery predict early mortality [59]. C-Tn levels and mortality increase with increasing complexity of cardiac surgery, such that the median c-Tn level rises progressively in patients undergoing isolated CABG with a single graft compared with 2 or more grafts [60].

Increased duration of cardiopulmonary bypass (CPB) and aortic cross clamping (X-Clamp) times in the elderly remain a concern. Longer CPB time is associated with increased incidence of cerebral, renal and coagulopathy, and greater X-Clamp time induces increased risk of myocardial damage, due to the lower efficiency of the physiological pathways of homeostasis. Furthermore, patients with severe CAD are more frequently affected by peripheral artery disease, which can increase the risk of postoperative ischemic complications with unfavorable outcomes, especially in elderly patients. A heart team approach including surgeons, cardiologists, anesthesiologists, internist and physiotherapists can be helpful to assess these elderly candidates and choose the best approach to treat AS [4]. For high-risk candidates, minimally invasive treatment options are desirable. Over the last decade, transcatheter aortic valve implantation (TAVI) has been identified as the standard of care for high-surgical risk patients, or for those considered inoperable by cardiac surgeons. The switch from SAVR to TAVI for elderly patients during recent years has led to a significant decrease of early mortality following AVR [61]. TAVI has demonstrated the potential to decrease the morbidity associated with standard SAVR owing to the avoidance of a median sternotomy, cardiopulmonary bypass and cardioplegic arrest.

Data from the recent randomized SURTAVI trial, comparing TAVI with PCI versus SAVR plus CABG in 332 patients, reported a 30-day mortality of 4.1% vs 3.7%, respectively, an incidence of stroke of 3.6% vs 4.3% and advanced acute kidney injury of 1.8% vs 3.7% [62]. The study excluded patients with SYNTAX score > 22, however, therefore it is not possible to extrapolate the outcomes from patients with more advanced CAD. Noteworthily the current guidelines for CAD recommend CABG for a high SYNTAX score and these patients could benefit from SAVR with CABG [63].

The German Aortic Valve Registry, an all-comers registry including 85 German centers, recently showed that the rate of in-hospital mortality for 26,618 patients undergoing isolated SAVR was 1.7%. The 30-day mortality in the 16,158 patients who underwent SAVR and CABG was significantly higher at 3.3%. In the SAVR plus CABG cohort stratified according to STS score risk, in 4044 patients in the intermediate category (STS score 4–8%), the in-hospital rate of mortality was 5.4%, the rate of disabling stroke was 2.4%, and need for new pacemaker or implantable cardioverter-defibrillator was 4.6% [64].

No unfavorable impact of CABG in combination with SAVR on long-term mortality compared with i-SAVR was reported in this updated systematic review and meta-analysis. The comparable long-term survival between the two treatments may support the rationale that CAD, although a recognized additional risk factor, when associated with aortic valve disease, probably does not result in increased long-term mortality when addressed with CABG. Among the 23 studies that reported follow-up data, there was a high range of mean follow-up times, varying from 2.1 to 6.5 years. Interestingly, when we narrowed the analysis to those studies that reported a maximum follow-up time of 10 years or more, again no differences were reported between the two treatments. Some authors have observed a long-term benefit of patients with concomitant CAD and AS undergoing CABG plus SAVR compared with patients who did not receive the CABG procedure at the time of SAVR [65]. The relief of AS, along with the addition of coronary revascularization, would increase coronary flow reserve and provide reversal remodeling as in patients with isolated AS who underwent i-SAVR. These factors would promote regression of left ventricular hypertrophy and increased coronary microcirculation, which are critical determinants of long-term survival [66].

In the meta-analysis, it is interesting to emphasize the validity and safety of the conventional surgical approach in elderly patients. Once the patient has gone through the postoperative period, where the CBAG + SAVR combination is associated with higher hospital mortality, long-term survival remains comparable between the two treatments. This finding has its clinical relevance and allows confirmation of the validity and safety of the conventional surgical approach, as well as that the associated CBAG has no negative clinical impact in the long term.

The incidence of new onset POAF increases with advancing age and the multifactorial pathophysiology has not been completely elucidated [67]. In this meta-analysis, no significant difference in postoperative AF incidence was identified between the two populations. One possible explanation for these data could be the higher incidence of POAF in elderly patients, regardless of the type of cardiac surgical procedure to which they undergo. In addition, severe aortic valve stenosis is a chronic disease that can lead to remodeling of the left ventricle with a decrease in diastolic compliance leading to increased left atrial volume and altered atrial function. Although CAD increases the risk of developing POAF [67], in this study, CABG was not associated with the development of POAF.

As age is an established risk factor for atherosclerotic disease, so there is an increased risk of aortic calcifications is expected in elderly patients [68]. Postoperative acute renal failure and dialysis appear to be lower in i-SAVR compared to SAVR plus CABG. A possible explanation is the increased rate of diabetes, hypertension, vascular disease, preoperative renal failure which are more represented in patients with CAD and longer CPB time in patients who underwent SAVR + CABG compared to i-SAVR [69–71]. CABG added to SAVR shows a nonsignificant trend toward a greater need for postoperative IABP compared to i-SAVR. Longer CPB and aortic X-clamp times, prolonged operative time, and peripheral vascular disease are predictive for postoperative IABP [72, 73]. These factors may explain why patients who underwent CABG in combination with SAVR had a higher incidence of postoperative IABP implantation. The meta-analysis shows that PMV was significantly associated with the SAVR + CABG surgical operation. Longer CPB time is reported to be an independent predictor of postoperative respiratory failure [74] and PMV (> 24 h) [75]. Since SAVR + CABG operation has a CPB time longer than i-SAVR, we can argue that this factor might be determinant in increasing the incidence of postoperative PMV in patients who received CABG added to SAVR. From the 17 studies that reported incidence of postoperative stroke, no significant differences emerged in patients undergoing i-SAVR compared to SAVR plus CABG. A plausible explanation for this finding is the pathophysiology of ischemic stroke post cardiac surgery. In patients undergoing aortic valve surgery, thromboembolism is likely attributable to aortic clamping and manipulation, as well as aortic valve decalcification, rather than to the duration of surgery [76, 77]. As cardiopulmonary bypass is required for both i-SAVR and CABG plus SAVR, similar thromboembolism rates would expect, since that both operations share the aortic manipulation.

## Limitations

The meta-analysis shares the limitation of meta-analyses of retrospective observational studies that can be affected from a risk of treatment allocation bias and unmeasured confounders. Moreover, the results of some studies included in the analysis are limited by a relatively small numbers of patients. In addition, it was not possible to extrapolate the incidence of incomplete myocardial revascularization data of those patients affected by aortic valve disease and CAD who were treated with only i-SAVR. In such a scenario, it is not possible to analyze the impact of untreated CAD in SAVR. Moreover, data related to survival outcome were not reported by each study included in the meta-analysis and therefore the reported pooled data on long-term survival needs to be interpreted with a word of caution. Finally, it was not possible to extrapolate patient selection criteria towards either conventional surgery or TAVI, and, therefore these results may be influenced by selection bias, as the elderly patients included in each study were likely fit for surgery. However, the large number of patients included in the meta-analysis may reduce the aforementioned bias and allows for robust results.

## Conclusions

In conclusion, in a meta-analysis of retrospective observational studies comparing early and long-term outcomes of patients undergoing aortic valve surgery, CABG in combination with SAVR is associated with a significantly higher incidence of 30-day mortality, whereas in the long-term follow-up the two treatments are comparable. Among the analyzed postoperative complications, CABG in combination with SAVR is associated with a higher incidence of acute renal failure, need for dialysis and PMV compared with i-SAVR. The incidence of postoperative stroke, POAF, need for IABP, re-thoracotomy for postoperative bleeding/tamponade, and length of stay were similar between the two treatments.

## Supplementary Information

Below is the link to the electronic supplementary material.

Supplementary file1 (PDF 1443 KB)
