# Supplementary material for: The early and long-term outcomes of coronary artery bypass grafting added to aortic valve replacement compared to isolated aortic valve replacement in elderly patients: a systematic review and meta-analysis
Source: Heart Vessels. 2022 May 9;37(10):1647–61. doi: 10.1007/s00380-022-02073-4 (PMC9399049; doi:10.1007/s00380-022-02073-4)
Supplement: Supplementary file 1 — Supplementary file1 (PDF 1443 KB) [file 380_2022_2073_MOESM1_ESM.pdf]

SUPPLEMENTAL MATERIALS

The early and long-term outcomes of coronary artery bypass grafting added to aortic valve replacement compared to isolated aortic valve replacement in elderly patients: A systematic review and meta-analysis.

Journal: Heart and Vessels

Stefano D'Alessandro MD, FECTS[1], Domenico Tuttolomondo MD[2], Gurmeet Singh MD[3], Daniel Hernandez-Vaquero MD[4], Claudia Pattuzzi MD[5], Alan Gallingani MD[5], Francesco Maestri MD[5], Francesco Nicolini MD, PhD [5,6] and Francesco Formica MD* [5,6].

[1] Cardiac Surgery Unit, San Gerardo Hospital, Monza, Italy.

[2] Cardiology Unit, University Hospital of Parma, Italy

[3] Department of Critical Care Medicine and Division of Cardiac Surgery, Mazankowski Alberta Heart Institute, University of Alberta, Canada.

[4] Cardiac Surgery Department, Hospital Universitario Central de Asturias, Oviedo, Spain

[5] Cardiac Surgery Unit, University Hospital of Parma, Italy

[6] Department of Medicine and Surgery, University of Parma

Corresponding Author:

Prof. Francesco Formica, MD
UOC Cardiochirurgia
Azienda Ospedaliera Universitaria di Parma
Via A. Gramsci, 14
43126 Parma, Italy
Tel.: +39 0521703278
E-mail: francescoformica.hs@gmail.com
francesco.formica@unipr.it

Supplemental Figures

Figure 1. PRISMA flow diagram……………………………………………………….page 3
Figures 2. Forrest plot for sensitivity analysis……………………………………….page 5
Figures 3. Funnel plot for publication bias……………………………………………..page 6

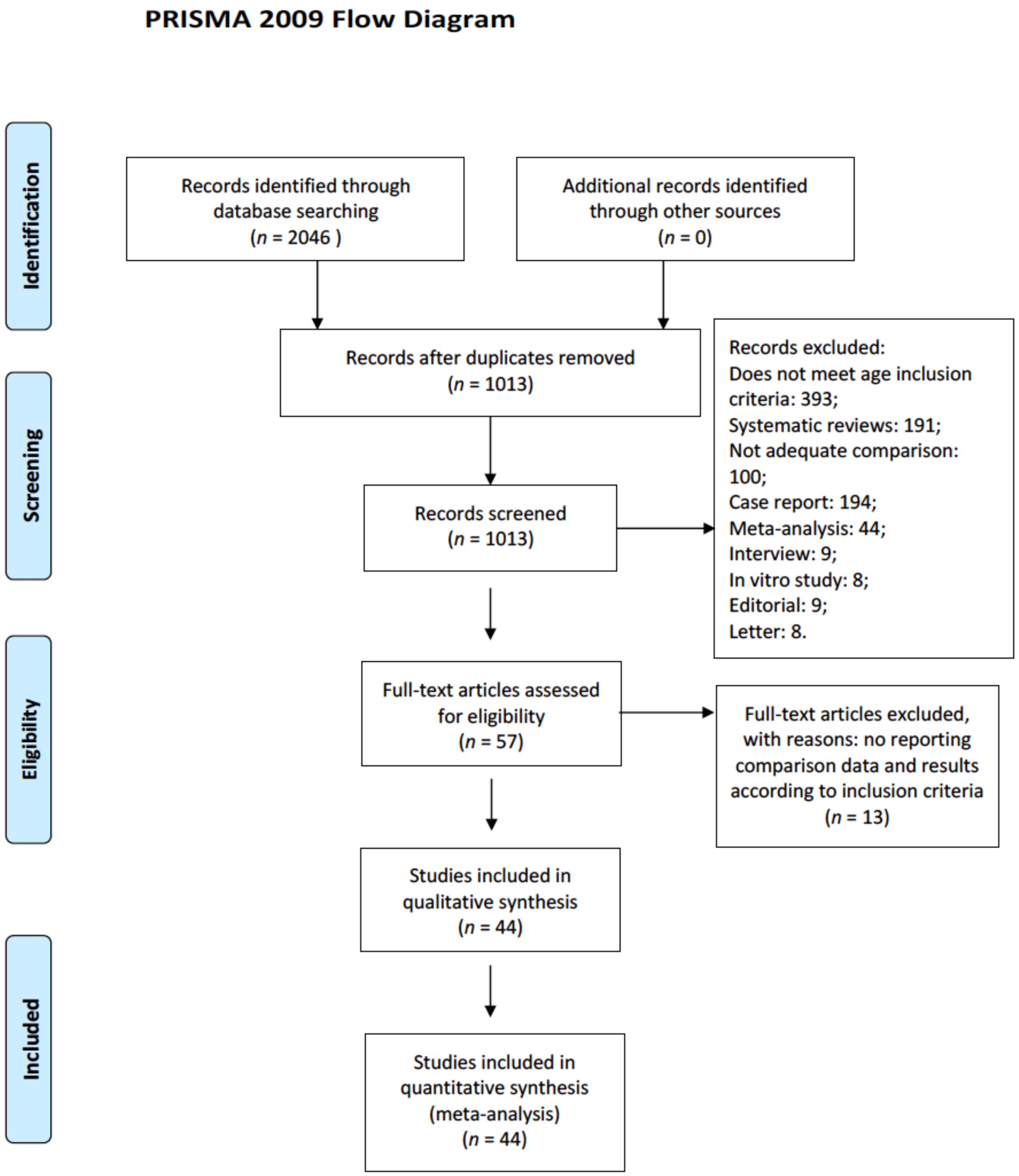

Figure 1. Study selection according to the Preferred Reporting Items for Systematic Reviews and Meta-Analyses (PRISMA), presented as a flow diagram.

	ES	95% CI	Sig.	N
Abel 2013	0.70	0.66 - 0.75	0.000	74120
Argawal 2015	0.64	0.56 - 0.74	0.000	12708
Bose 2007	0.70	0.66 - 0.75	0.000	74432
Brunvand 2001	0.70	0.66 - 0.75	0.000	74406
Budniak 2014	0.70	0.66 - 0.75	0.000	74460
Chiappini 2004	0.71	0.66 - 0.75	0.000	74385
Davis 2014	0.70	0.66 - 0.75	0.000	74437
Dell'Amore 2011	0.70	0.66 - 0.75	0.000	74215
Dimagli 2020	0.71	0.66 - 0.76	0.000	72788
Ennker 2001	0.71	0.66 - 0.75	0.000	74386
Ennker 2018	0.71	0.66 - 0.76	0.000	73794
Filsoufi 2008	0.70	0.66 - 0.75	0.000	74308
Florath 2010	0.70	0.66 - 0.75	0.000	74007
Folkmann 2010	0.70	0.66 - 0.75	0.000	74346
Formica 2020	0.70	0.66 - 0.75	0.000	74098
Grau 2014	0.70	0.66 - 0.75	0.000	74307
Harris 2013	0.70	0.66 - 0.75	0.000	74383
Ho 2014	0.70	0.66 - 0.75	0.000	74368
Huber 2007	0.70	0.66 - 0.75	0.000	74425
Kamiya 2014	0.70	0.66 - 0.75	0.000	74162
Kesavan 2011	0.70	0.66 - 0.75	0.000	74227
Kolh 2007	0.71	0.66 - 0.76	0.000	74280
Krane 2011	0.70	0.66 - 0.75	0.000	73900
Kuo 2017	0.70	0.66 - 0.75	0.000	74122
Lam 2004	0.70	0.66 - 0.75	0.000	74442
Langanay 2012	0.71	0.66 - 0.76	0.000	73355
Likosky 2009	0.70	0.66 - 0.75	0.000	73110
Litmathe 2003	0.70	0.66 - 0.75	0.000	74217
Maillet 2009	0.71	0.66 - 0.75	0.000	74416
Maslow 2010	0.70	0.66 - 0.75	0.000	74239
Melby 2007	0.70	0.66 - 0.75	0.000	74255
Mitchell 2013	0.70	0.66 - 0.75	0.000	74433
Ngaage 2007	0.70	0.66 - 0.75	0.000	74313
Nikolaidis 2011	0.70	0.66 - 0.75	0.000	74155
Raja 2003	0.70	0.66 - 0.75	0.000	74318
Roberts 2007	0.70	0.66 - 0.75	0.000	74304
Salsano 2016	0.70	0.66 - 0.75	0.000	74445
Sasaki 2013	0.70	0.66 - 0.75	0.000	74343
Takagi 2020	0.70	0.66 - 0.75	0.000	74471
Thulin 2000	0.70	0.66 - 0.75	0.000	74281
Unic 2005	0.70	0.66 - 0.75	0.000	74260
Urso 2007	0.70	0.66 - 0.75	0.000	74400
Wang 2016	0.70	0.66 - 0.75	0.000	74303
Yamane 2011	0.70	0.66 - 0.75	0.000	74376

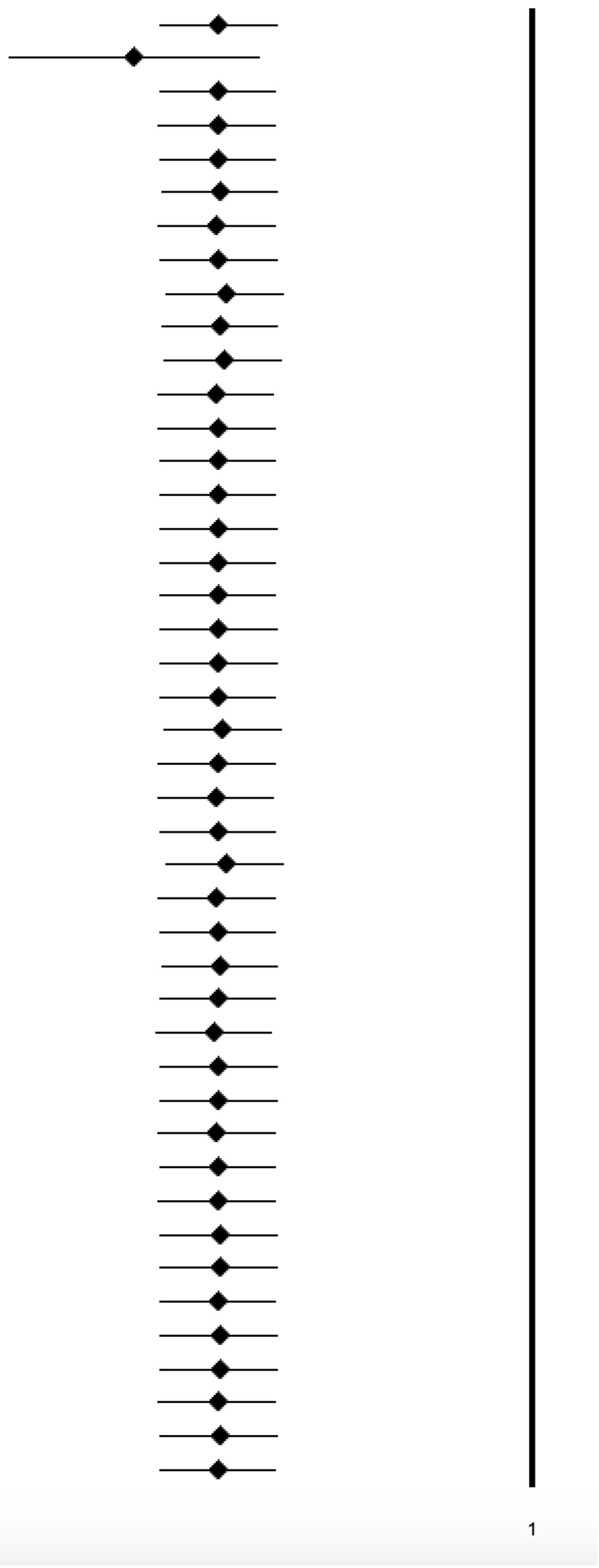

Figure 2A. Forest plot of the leave-one-out analysis comparing the early-term effect of coronary artery bypass grafting combined with aortic valve replacement versus isolated aortic valve replacement . *ES: odd ratio.*

	ES	95% CI	Sig.	N
Brunvand 2001	0.95	0.87 - 1.03	0.234	6770
Chiappini 2004	0.95	0.87 - 1.04	0.266	6749
Dell'Amore 2011	0.96	0.88 - 1.04	0.298	6579
Ennker 2018	0.95	0.86 - 1.04	0.260	6158
Florath 2010	0.98	0.91 - 1.05	0.575	6371
Folkmann 2010	0.95	0.87 - 1.04	0.266	6710
Formica 2020	0.95	0.87 - 1.04	0.267	6462
Grau 2014	0.95	0.87 - 1.03	0.234	6671
Huber 2007	0.95	0.88 - 1.04	0.273	6789
Kesavan 2011	0.95	0.87 - 1.04	0.290	6591
Krane 2011	0.97	0.90 - 1.05	0.491	6264
Kuo 2017	0.94	0.87 - 1.02	0.135	6486
Likosky 2009	0.94	0.85 - 1.03	0.168	5474
Maslow 2010	0.94	0.86 - 1.03	0.178	6603
Melby 2007	0.95	0.88 - 1.01	0.116	6619
Nikolaidis 2011	0.96	0.88 - 1.04	0.314	6519
Roberts 2007	0.94	0.87 - 1.02	0.132	6668
Salsano 2016	0.95	0.87 - 1.04	0.262	6807
Sasaki 2013	0.95	0.88 - 1.04	0.274	6707
Takagi 2020	0.95	0.88 - 1.03	0.252	6835
Thulin 2000	0.95	0.87 - 1.03	0.224	6645
Wang 2016	0.95	0.87 - 1.04	0.268	6667

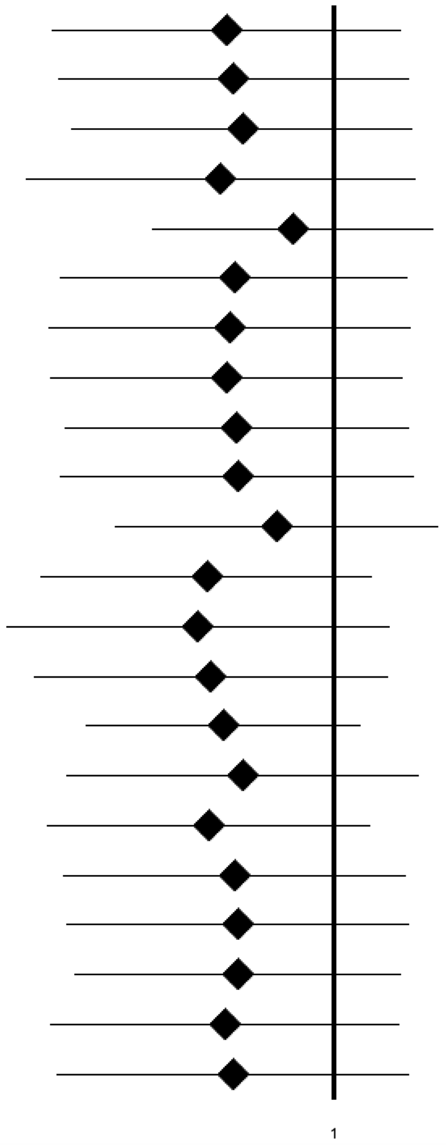

Figure 2B. Forest plot of the leave-one-out analysis comparing the long-term effect of coronary artery bypass grafting combined with aortic valve replacement versus isolated aortic valve replacement . *ES: odd ratio.*

FIGURE 3.

Funnel plots for pubblication bias assesment.

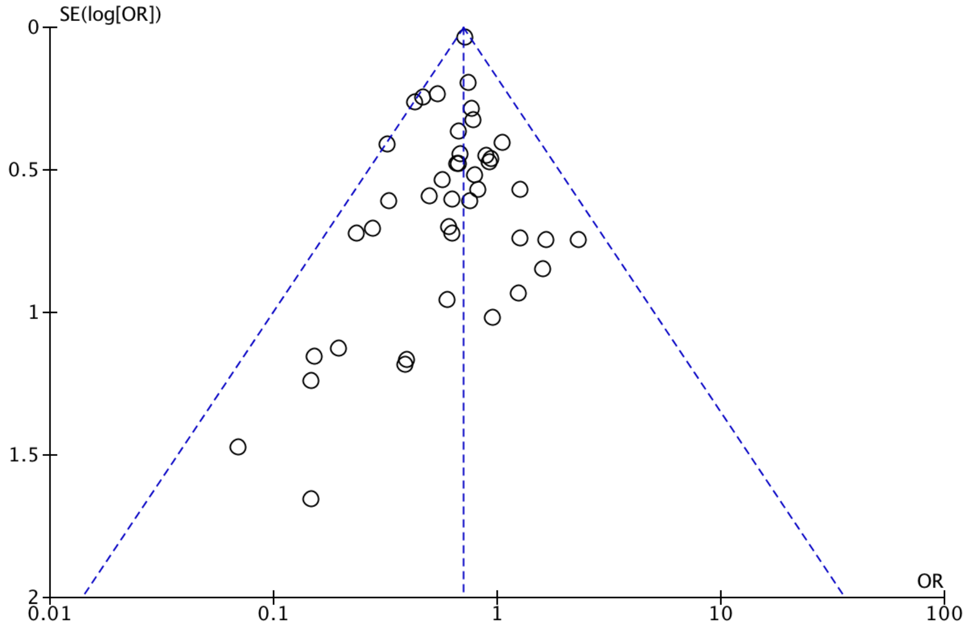

A. No publication bias was reported related to the early mortality. Egger's linear regression test: p=0.06; Begg and Mazumdar's test: p=0.15.

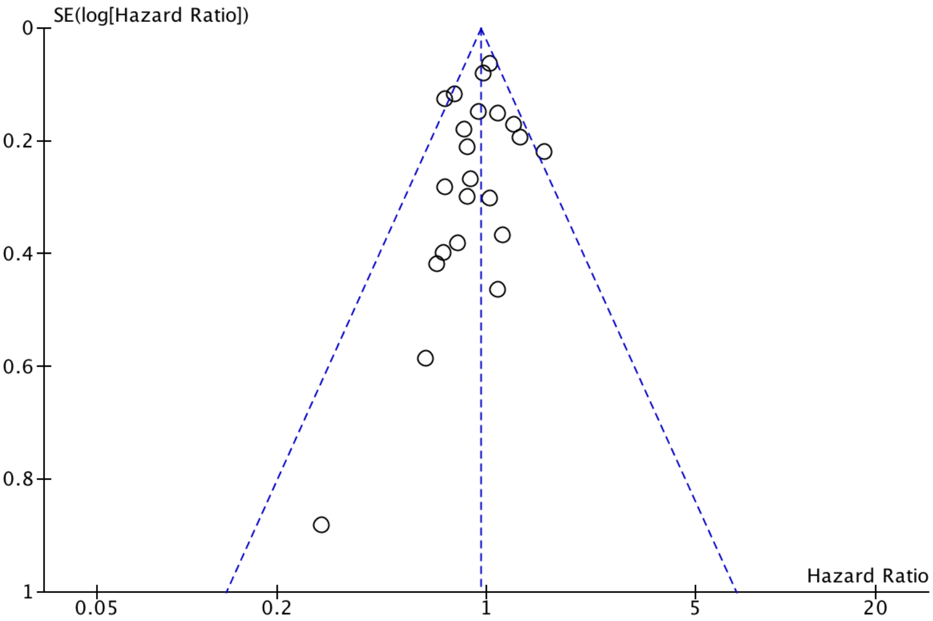

B. No publication bias was reported related to the long-term survival. Egger's linear regression test: p = 0.27; Begg and Mazumdar's linear regression test: p = 0.19.

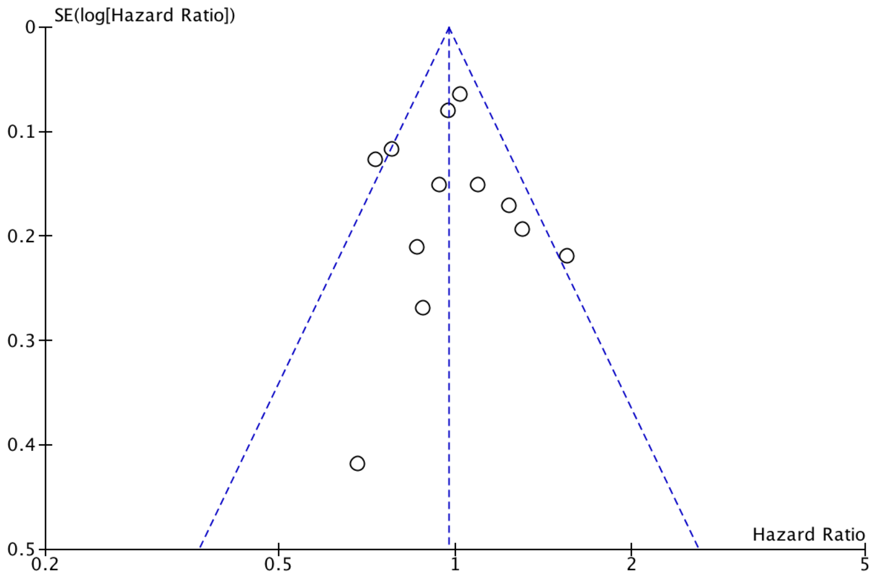

C. No publication bias was reported related to long-term survival of studies reporting a maximum follow-up of ten years or more. Egger's linear regression test: p=0.83; Begg and Mazumdar's test: p=0.90.

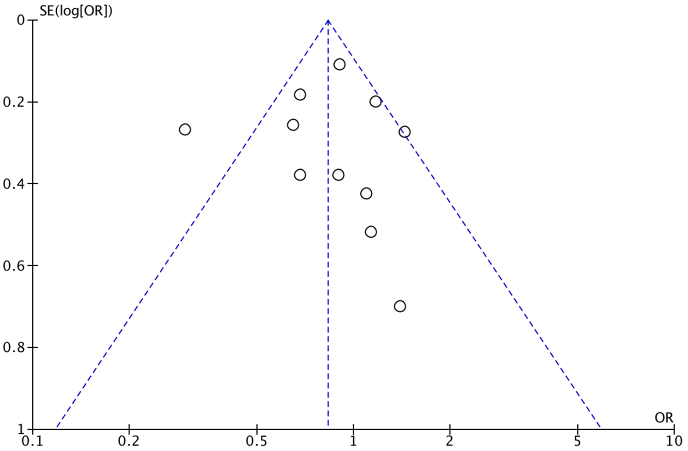

D. No publication bias was reported related to postoperative atrial fibrillation. Egger's linear regression test: p=0.96; Begg and Mazumdar's test: p=0.58.

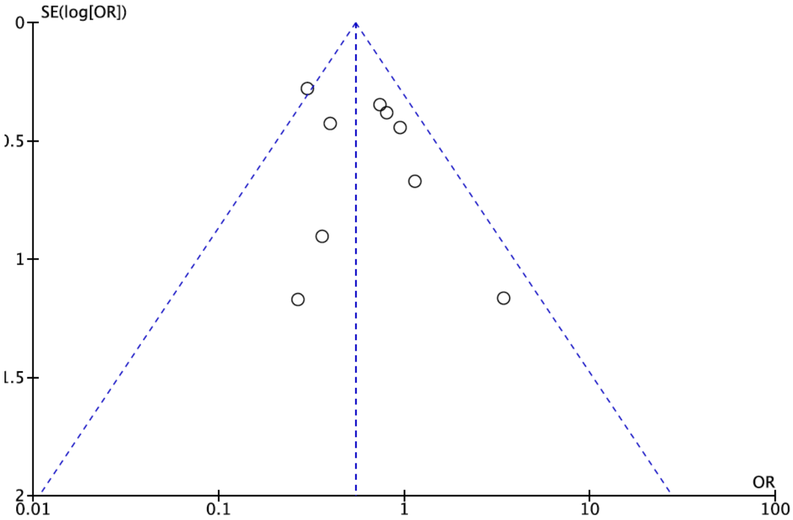

E. No publication bias was reported related to postoperative acute renal failure. Egger's linear regression test: p=0.33; Begg and Mazumdar's test: p=0.29.

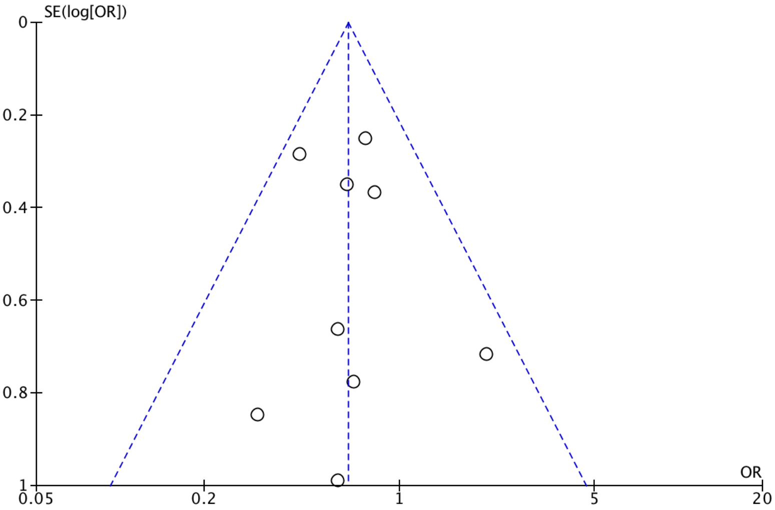

F. No publication bias was reported related to need for postoperative dyalisis. Egger's linear regression test: p = 0.53; Begg and Mazumdar's test: p > 0.99.

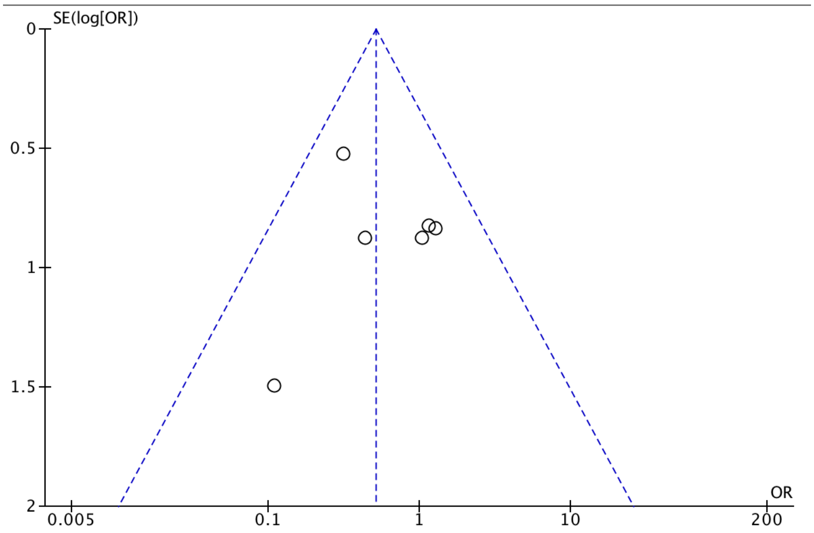

G. No publication bias was reported related to postoperative intra-aortic balloon pump usage. Egger's linear regression test: p = 0.67; Begg and Mazumdar's test: p = 0.88.

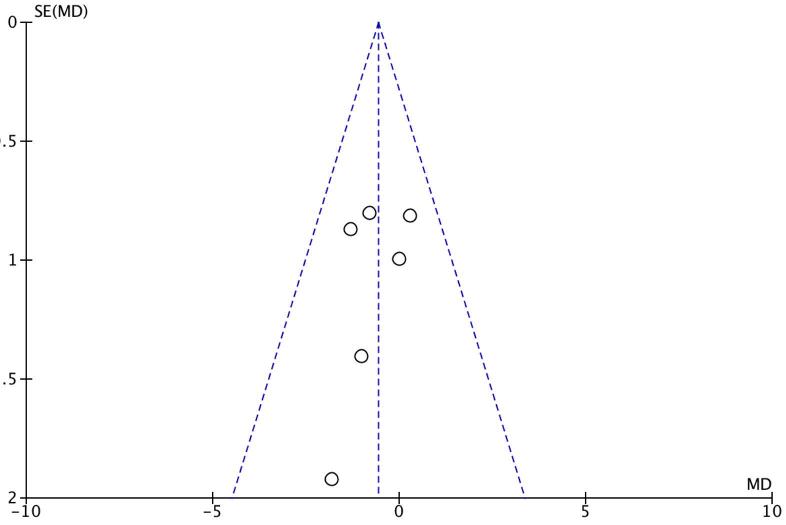

H. No publication bias was reported related to length of hospital stay. Egger's linear regression test: p=0.42; Begg and Mazumdar's test: p=0.32.

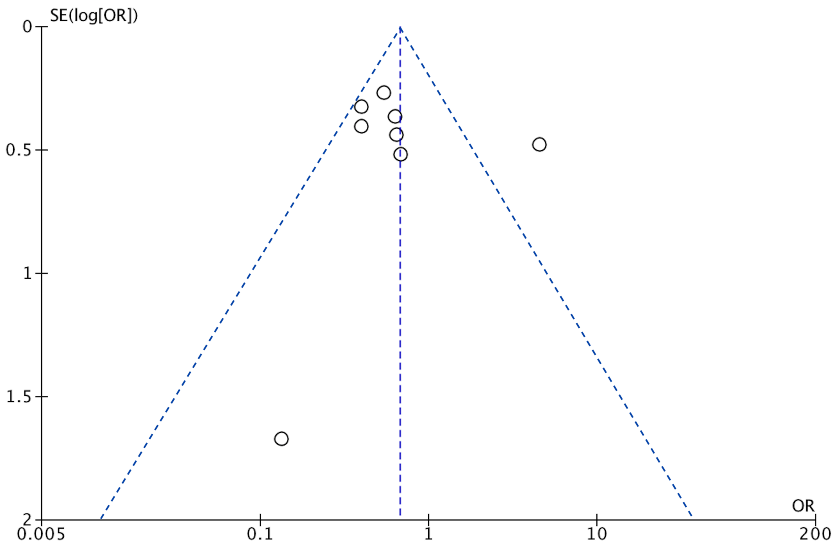

I. No publication bias was reported related to prolonged mechanical ventilation (> 48 hours). Egger's linear regression test: p = 0.76; Begg and Mazumdar's test: p = 0.21.

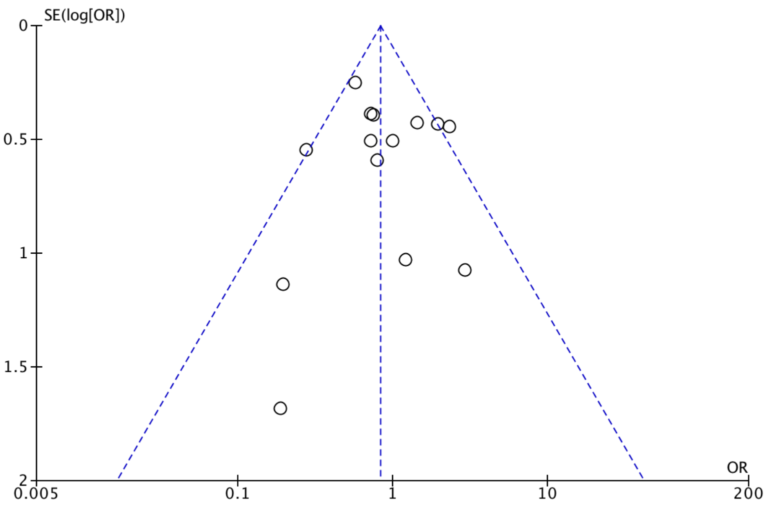

L. No publication bias was reported related to re-thoracothomy for postoperative bleeding/tamponade. Egger's linear regression test: p = 0.93; Begg and Mazumdar's test: p = 0.89.

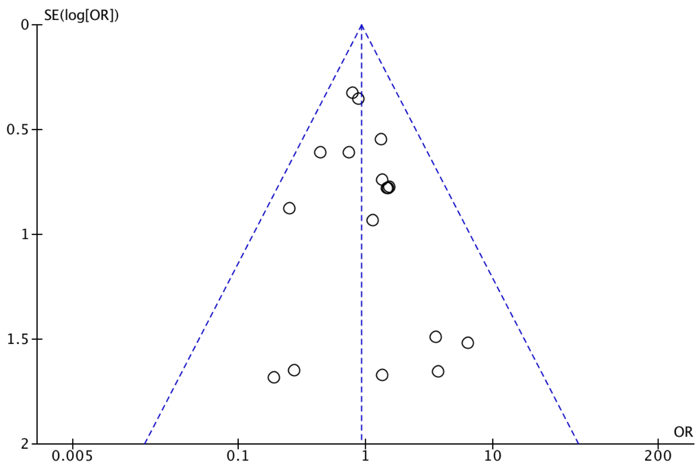

M. No publication bias was reported related to postoperative stroke. Egger's linear regression test: p=0.89; Begg and Mazumdar's test: p=0.78.

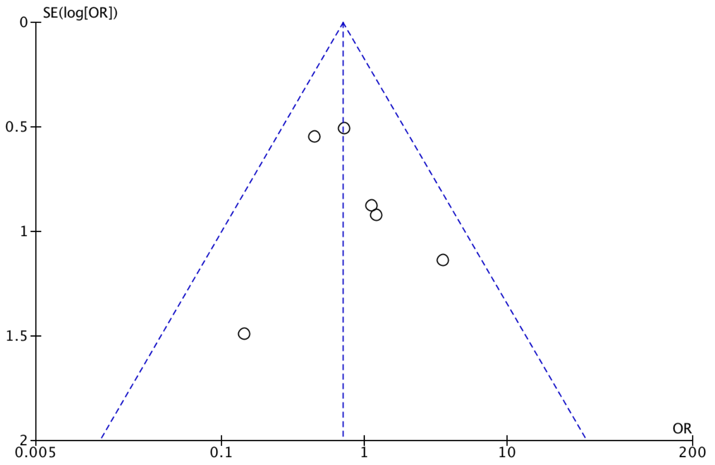

N. Funnel plot to assess publication bias. No publication bias was reported related to postoperative pneumonia. Egger's linear regression test: p = 0.64; Begg and Mazumdar's test: p = 0.57.
